# Synthesis of the Sex Pheromone of the Tea Tussock Moth Based on a Resource Chemistry Strategy

**DOI:** 10.3390/molecules23061347

**Published:** 2018-06-04

**Authors:** Hong-Li Zhang, Zhi-Feng Sun, Lu-Nan Zhou, Lu Liu, Tao Zhang, Zhen-Ting Du

**Affiliations:** 1 State Key Laboratory of Crop Stress Biology in Arid Areas, Northwest A&F University, Yangling 712100, China; honglizhang@126.com; 2 Shaanxi Key Laboratory for Catalysis, College of Chemical and Environment Science, Shaanxi University of Technology, Hanzhong 723001, China; sunzhifeng2018@163.com; 3 College of Chemistry and Pharmacy, Northwest A&F University, Yangling 712100, China; Zhou18792419302@163.com (L.-N.Z.); m18706733265@163.com (L.L.); 4 Key Laboratory of Botanical Pesticide R&D in Shaanxi Province, Yangling 712100, China

**Keywords:** insect sex pheromone, tea tussock moth, total synthesis, resource chemistry

## Abstract

Synthesis of the sex pheromone of the tea tussock moth in 33% overall yield over 10 steps was achieved. Moreover, the chiral pool concept was applied in the asymmetric synthesis. The synthesis used a chemical available on a large-scale from recycling of wastewater from the steroid industry. The carbon skeleton was constructed using the C4+C5+C8 strategy. Based on this strategy, the original chiral center was totally retained.

## 1. Introduction

Today, safer agricultural produce demands that farming procedures be performed in a greener way. In this context, integrated pest management (IPM) is proposed, which is a broad-based approach that integrates practices for the economic control of pests. Among them, using low-dose pheromones to control pest has been accepted by more and more agricutural providers. In East Asia, a kind of pest called tea tussock moth (*Euproctis pseudoconspersa*) causes huge destruction in tea orchids. The bit leaves may fall off and decrease the gross product. If this kind of pest was controlled by pheromone, the tea leaves could be provided better than before. The main component of the moth’s sex pheromone was first reported by Wakamura as 10,14-dimethyl-l-pentadecyl isobutyrate [1,2]. Then, a field attraction attempt was tested using crude extract from females and synthetic pheromones [3], and the result revealed that both *S* and *R* configurations have similar luring activities. As continuation of our interest in green agrochemicals [4,5,6,7], a synthesis of insect pheromone **1** based on a resource chemistry strategy was envisioned. As a matter of fact, there are several synthetic approaches to this compound in the literature. Ichikawa [8] and Zhao [2] synthesized the tussock moth pheromone (*R)*-**1** from (*S*)-citronellol or its corresponding bromide. Very recently, a synthesis of tussock pheromone based on a protective-group-free strategy [9] has been reported by our group, in which the Evans’ template was adopted to control the chirality.

Some 4000 tons of sapogenin are produced every year by the Chinese steroid industry. If a new H_2_O_2_ oxidation procedure were to be applied to pregna-16,20-diol, a large quantity of (5*S*)-3-hydroxy-5-methyltetrahydro-2*H*-pyran-2-one (**2**) could become available, and 1000 tons of chiral material could be recycled. Tian et al. developed a toolkit of chiral building blocks from this resource chemical [10] and used it to achieve several syntheses of natural compounds [6,11,12,13]. He coined the term “resource chemical”, which refers to large-scale and useful substances. Herein, we discuss the synthesis of the pheromone of the tea tussock moth based on this resource chemical.

## 2. Results and Discussions

**Scheme 1:**
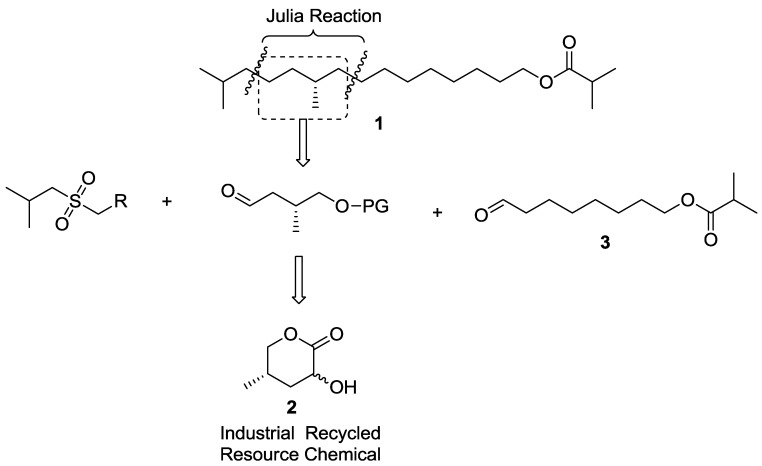
Retrosynthesis of compound **1** based on Julia olefination and a resource-chemical.

As Scheme 1 shows, the retrosynthesis was based on two Julia coupling reactions, namely a C4+C5+C8 strategy. The left hand C4 synthon was easily obtained, and the right C8 subunit was a protected aldehyde **3**. The middle unit can be derived from a chiral methyl aldehyde, which is a large-scale resource chemical derivative.

**Scheme 2:**
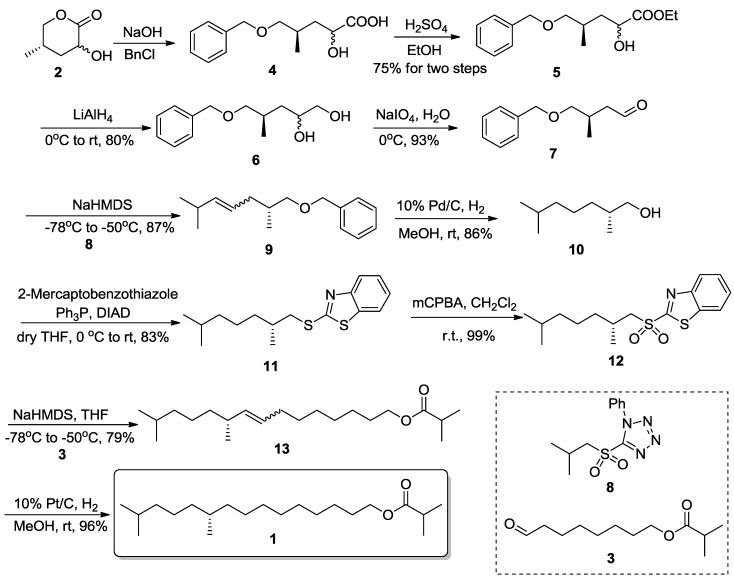
Synthesis of (*S*)-**1** through a double Julia approach.

As Scheme 2 shows, the synthesis commenced from chiral lactone **2** which was ring-opened [14,15] under basic conditions and subjected to selective benzylation to give compound **4**. In practice it proved laborious to purify the very polar hydroxyacid, so it was used directly without further purification. To reduce the carboxylic acid effectively, the ethyl ester **5** could be obtained in 75% yield for two steps under acid-catalyzed esterification conditions [11], but according to the ^1^H-NMR and ^13^C-NMR spectra (supplementary material), it could be observed that the hydroxyl group in the 2-position was partially epimerized. We suspect this resulted from the basic conditions used. We could observe some small peaks and high peaks with a height ratio of *c.a.* 1:3. Fortunately, the chirality of the desired methyl group didn’t matter. The subsequent reduction by LiAlH_4_ gave the vicinal diol **6** in 80% yield, which is a good substrate for an aldehyde. Therefore, the chiral aldehyde (*R*)-4-(benzyloxy)-3-methylbutanal **7** could be produced in 93% yield after a conventional oxidative diol cleavage using NaIO_4_ in aqueous solvent. Several preparation methods of compound **7** that can be found in the literature. Aside from Wei’s route [11], the rest require more chemical operations [16], an expensive chiral auxiliary such as the Evans template [17,18,19] or poisonous cyanide [20]. The e.e. was determined by comparison of the optical rotation value [α]_D_^25^ + 10.6 (*c* 0.7, CHCl_3_); lit. [17] [α]_D_^20^ + 10.4 (*c* 0.7, CHCl_3_). Next, at −78–−50 °C, a Julia-Kocienski reaction was performed between 5-(isobutylsulfonyl)-1-phenyl-1*H*-tetrazole (**8**), which was prepared according to the literature [21] and the chiral aldehyde **7**, affording the coupling product **9** in 87% yield (*E/Z* ratio of 1:1). The subsequent hydrogenation of the C=C double bond and removal of the benzyl protecting group was achieved in one-pot in 86% yield. (*R*)-2,6-dimethylheptan-1-ol (**10**) was converted into a Julia sulfone reagent **12** through the Mitsunobu protocol [22], followed by an oxidative reaction (83% and 99% yield, respectively). A Julia-Lythgoe reaction between aldehyde **3** and sulfone **12** gave the corresponding alkene product **13** in 79% yield with an *E*/*Z* ratio of 2:1. To prevent the isomerization and racemization of the chiral center, alkene **13** was subjected to Pt/C catalytic hydrogenation [23], giving (*S*)-**1** in 99% yield. All the analytic data for synthetic compound **1** are in accordance with that reported in the literature [8,9].

**Scheme 3:**
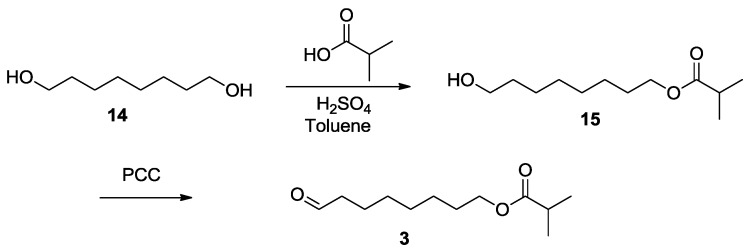
Synthesis of compound **3**.

As Scheme 3 shows, similar to the literature [9], octane-1,8-diol was esterified selectively with 70% yield using a stoichiometric amount of isobutyric acid at presence of catalytic sulfuric acid in toluene [24]. Then, a pyridinium chlorochromate (PCC) [25] oxidation was applied, and the aldehyde **3** was produced in 90% yield (Scheme 3).

## 3. Experimental Section

### 3.1. General Methods

THF was distilled from sodium/benzophenone and CH_2_Cl_2_ was distilled from CaH_2_ before use. Reactions were monitored by thin-layer chromatography (TLC) on glass plates coated with silica gel with fluorescent indicator. Flash chromatography was performed on silica gel (200–300) with petroleum/EtOAc as the eluent. Optical rotations were measured on a polarimeter with a sodium lamp. HRMS spectra were measured on a LCMS-IT-TOF or LTQ-Orbitrap-XL apparatus. IR spectra were recorded using a Fourier transform infrared spectrometer. NMR spectra were recorded on a AC-500 MHz instrument (Bruker, Madison, WI, USA). Chemical shifts were reported in δ (ppm) and referenced to an internal TMS standard for ^1^H-NMR and CDCl_3_ (77.16 ppm) for ^13^C- NMR (Supplementary material).

### 3.2. Ethyl (4R)-5-(Benzyloxy)-2-Hydroxy-4-Methylpentanoate (***5***)

The raw material **2** was obtained from the Pharmaceutical Factory of Shaanxi Academy of Science, Yangling, China as a strong basic, aqueous solution. If the water was removed, a large-scale raw (70–75% purity) brown solid could be obtained. In this paper, this solution was acidified to pH = 1, filtered, and the filtrate was extracted with ethyl acetate to afford crude **2**. Compound **2** was purified through column chromatography. Because the straight separation of **2** from proved laborious, the raw lactone **2** was used directly sometimes to save time.

To purified lactone **2** (6.5 g, 50 mmol), toluene (100 mL) and powdered NaOH (8 g, 200 mmol) were added. After the mixture was refluxed for 6 h, at the same time, the produced water was removed through a Dean-Stark trap, and then BnCl (15.8 g, 125 mmol) was added in 3–5 batches, and the reaction mixture was allowed to react for another 12 h. Then, water (100 mL) was added, and the aqueous phase was extracted with Et_2_O (70 mL × 3). The organic phases were discarded, and the aqueous phase was acidified with concentrated hydrochloric acid to pH = 1. The resulting aqueous phase was extracted with EtOAc (70 mL × 3), and the combined organic layers were dried over MgSO_4_, filtered, and concentrated to give the crude acid without further purification. The above crude acid was dissolved in EtOH (150 mL), and then H_2_SO_4_ (5.00 mL, 98%) was added dropwise. After the mixture was refluxed for 4 h, it was monitored by TLC. Then, the reaction was dispensed into EtOAc (250 mL), and washed with water, saturated NaHCO_3_, and brine. The solvent was evaporated, and the residue was purified through column chromatography to give the ester **5** with partly epimerized hydroxyl group 8.9 g as a yellowish oil. IR (film) ν_max_ 3450, 2958, 2851, 2359, 1737, 1458, 1211, 1092, 740, 701 cm^−1^; ^1^H-NMR (500 MHz, CDCl_3_) δ 7.33–7.37 (m, 5H), 4.22−4.35 (m, 3H), 3.30−3.40 (m, 1H), 3.31−3.38 (m, 2H), 2.04−2.14 (m, 1H), 1.62−1.77 (m, 2H), 1.29 (t, *J* = 7.1 Hz, 2H), 1.05 (d, *J* = 6.8 Hz, 3H); ^13^C-NMR (125 MHz, CDCl_3_) δ 175.4, 138.2, 130.2, 128.4, 127.6, 75.8, 73.1, 69.3, 61.5 39.3, 30.6, 16.8, 14.2.

### 3.3. (R)-4-(Benzyloxy)-3-Methylbutanal (***7***)

To a suspension of LAH (1.11 g, 30.0 mmol) in THF (45 mL), a solution of ester **5** (5.2 g, 19.5 mmol) in THF (10 mL) was added dropwise in an ice bath. The reaction mixture was warmed to r.t after being stirred for 3 h, followed by another stirring for 10 h. The resulting mixture was carefully quenched with water (1 mL) and aqueous NaOH (5%, 2 mL), and the precipitated salt was filtered. The cake was washed with THF (30 mL), combined with the filtrate, and concentrated to give the crude diol without further purification. From the crude NMR, we could see there were two diastereomers in ca 1:3. At 0 °C, to diol **6** (1.2g 5 mmol) in water (20 mL), sulfuric acid was added to pH = 6. Then NaIO_4_ (1.6 g, 7.5 mmol) was added portionwise and the resulting mixture was stirred for 1.5 h. After the reaction was complete, the mixture was extracted with Et_2_O (30 mL × 3), and the combined organic layers were washed with brine. The extract was dried, filtrated, and concentrated to give the aldehyde **7** as a colorless oil with 87% yield in two steps. [α]_D_^25^ +10.6 (*c* 0.7, CHCl_3_); ^1^H-NMR (500 MHz, CDCl_3_) δ: 9.80 (t, 1H, *J* = 3.2Hz), 7.32–7.41 (m, 5H), 4.53 (s, 2H), 3.42 (dd, *J* = 9.1, 5.2 Hz, 1H), 3.26 (dd, *J* = 9.1, 7.7 Hz, 1H), 2.56 (ddd, *J* = 16.2, 6.3, 2.3 Hz, 1H), 2.30–2.44 (m, 1H), 2.28 (ddd, *J* = 16.2, 7.0, 2.1 Hz, 1H), 0.99 (d, *J* = 6.8 Hz, 3H); ^13^C-NMR (125 MHz, CDCl_3_) δ: 202.4, 138.3, 128.4, 127.6, 127.6, 74.9, 73.1, 48.5, 29.2, 17.1.

### 3.4. (2R)-(((2,6-Dimethylhept-4-en-1-yl)oxy)Methyl)Benzene (***9***)

To a stirred solution of compound **8** (2.4 g, 8.9 mmol) in THF (30.0 mL) at –78 °C under argon, NaHMDS (2.0 M in THF, 5.60 mL, 11.3 mmol) was added dropwise. After 30 min, a solution of compound **7** (1.4 g, 7.26mmol) in THF (10.0 mL) was added dropwise. Upon stirring at −78 °C for 2 h, the reaction was warmed to −50 °C overnight. After the reaction was quenched with saturated aqueous NH_4_Cl at −50 °C, it was extracted with ethyl acetate, washed with brine, dried over anhydrous Na_2_SO_4_, concentrated, and purified by flash chromatography on silica gel (hexanes: ethyl acetate = 20:1) to give *E*/*Z* ca. 1:1 mixture of compound **9** as a pale-yellow oil (1.9 g, 87%). [α]_D_^25^ + 1.32 (*c* 2.5, CHCl_3_). ^1^H-NMR (500 MHz, CDCl_3_) δ 7.34–7.25 (m, 5H), 5.37–5.22 (m, 2H), 4.5 (d, *J* = 2.5 Hz, 2H), 3.36–3.23 (m, 2H), 2.6–2.11 (m, 2H), 1.96–1.79 (m, 2H), 0.96–0.9 (m, 9H); ^13^C-NMR (125 MHz, CDCl_3_) δ 139.4, 138.7, 128.3, 127.6, 127.6, 127.4, 125.2, 124.8, 75.5, 75.4, 73.0, 73.0, 36.6, 34.0, 33.8, 31.3, 31.1, 26.4, 23.1, 23.1, 22.7, 22.7, 17.0, 16.8.

### 3.5. (R)-2,6-dimethylheptan-1-ol (***10***)

To a solution of ether **9** (1.6 g, 6.89 mmol) in methanol (50 mL), Pd/C (10%, 0.3 g) was added and the atmosphere was exchanged with H_2_. The reaction mixture was stirred for 24 h and monitored by TLC. After completion, the catalyst was filtered through a Celite pad, and the filtrate was evaporated, giving compound **10** after flash column purification as a pale-yellow oil (0.85 g, 86%). [α]_D_^25^ + 4.68 (*c* 2.4, CHCl_3_). ^1^H-NMR (500 MHz, CDCl_3_) δ 3.53-3.49 (m, 1H), 3.44-3.39 (m, 1H), 1.61 (s, 1H), 1.56–1.51 (m, 1H), 1.36–1.08 (m, 7H), 0.92 (d, *J* = 6.6 Hz, 3H), 0.87 (d, *J* = 6.4 Hz, 6H); ^13^C-NMR (125 MHz, CDCl_3_) δ 68.4, 35.8, 33.4, 27.9, 24.7, 22.7, 22.6, 16.6.

### 3.6. (R)-2-((2,6-Dimethylheptyl)thio)Benzo[d]Thiazole (***11***)

Under argon, to a flask with (*R*)-2,6-dimethylheptan-1-ol **10** (0.7 g, 4.85mmol), THF (25 mL), benzo[*d*]thiazole-2-thiol (1.0 g, 6.0 mmol), and TPP (1.5 g, 6.0 mmol) were added. To this mixture, DIAD (1.1 mL, 6.0mmol) was added dropwise at 0 °C. After the reaction was completed, the volatile was evaporated, and the residue was purified through column chromatography to give compound **11** (1.2 g, 83% yield). [α]_D_^25^ –3.63 (*c* 3.1, CHCl_3_). ^1^H-NMR (500 MHz, CDCl_3_) δ 7.9 (d, *J* = 8.1 Hz, 1H), 7.79 (d, *J* = 8 Hz, 1H), 7.45 (t, *J* = 7.3 Hz, 1H), 7.33 (t, *J* = 7.3 Hz, 1H), 3.48–3.22 (m, 2H), 2.01–1.95 (m, 1H), 1.6–1.3 (m, 5H), 1.24–1.2 (m, 2H), 1.12 (d, *J* = 6.7Hz, 3H), 0.92 (d, *J* = 6.6 Hz, 6H); ^13^C-NMR (125 MHz, CDCl_3_) δ 167.79, 153.38, 135.19, 126.0, 124.09, 121.45, 120.91, 40.78, 39.06, 36.35, 33.3, 27.95, 24.69, 22.71, 22.6, 19.4. HRMS (ESI) *m*/*z* calcd. for C_16_H_24_NO_2_S_2_^+^ (M + H)^+^: 326.1248, found 326.1245.

### 3.7. (R)-2-((2,6-Dimethylheptyl)Sulfonyl)Benzo[d]Thiazole (***12***)

To a solution of compound **11** (1.0 g, 3.61mmol) in CH_2_Cl_2_ (35 mL), *m*CPBA (4.7 g, 70%, 5.6 eq) was added, and the reaction mixture was stirred for 12 h at r.t. Then, saturated Na_2_S_2_O_3_ was added to quench the reaction, and the reaction was neutralized using saturated Na_2_CO_3_ and extracted with CH_2_Cl_2_. The combined organic layers were washed with brine, and the extract was dried over MgSO_4_, filtrated, and concentrated to give compound **12** as a colorless oil with 99% yield. [α]_D_^25^ − 2.99 (*c* 2.6, CHCl_3_). ^1^H-NMR (500 MHz, CDCl_3_) δ 8.21 (d, *J* = 8.0 Hz, 1H), 8.01 (d, *J* = 7.6 Hz, 1H), 7.66–7.58 (m, 2H), 3.56 (dd, *J* = 14.3, 4.7 Hz, 1H), 3.35 (dd, *J* = 14.3, 8.0 Hz, 1H), 2.31–2.25 (m, 1H), 1.49–1.43 (m, 2H), 1.34–1.25 (m, 3H), 1.14 (d, *J* = 6.7 Hz, 3H), 1.11–1.06 (m, 2H), 0.82(d, *J* = 6.5, 3H), 0.81(d, *J* = 6.5 Hz, 3H); ^13^C-NMR (125 MHz, CDCl_3_) δ 166.83, 152.76, 136.79, 127.98, 127.64, 125.46, 122.37, 60.83, 38.7, 36.89, 28.57, 27.84, 24.08, 22.57, 22.49, 19.91. HRMS (ESI) *m*/*z* calcd. for C_16_H_24_NO_2_S_2_^+^ (M + H)^+^: 326.1248, found 326.1245.

### 3.8. 8-Hydroxyoctyl Isobutyrate ***15***

To a solution of octane-1,8-diol (7.3 g, 50 mmol) and isobutyric acid (4.2 g, 48 mmol) in toluene (100 mL), four drops of concentrated sulfuric acid were added, and the reaction mixture was warmed to 75 °C for 20 h. The reaction mixture was diluted using ethyl acetate (200 mL) and washed with water, NaHCO_3_, and brine, then dried over MgSO_4_. The solvent was evaporated, and the residue was purified through column chromatography to give compound **15** (8.8 g, 85%) as a colorless oil. ^1^H- NMR (500 MHz, CDCl_3_) δ 4.04 (t, *J* = 6.7 Hz, 2H), 3.62 (t, *J* = 6.6 Hz, 2H), 2.55–2.49 (m, 1H), 2.03 (s, 1H), 1.62–1.52 (m, 4H), 1.32 (s, 8H), 1.15 (d, *J* = 7.0 Hz, 6H); ^13^C-NMR (125 MHz, CDCl_3_) δ 177.26, 64.31, 62.93, 34.02, 32.70, 29.24, 29.15, 28.59, 25.79, 25.62, 18.97.

### 3.9. 8-Oxo-Octyl Isobutyrate (***3***)

To a solution of 8-hydroxyoctyl isobutyrate **15** (2.8 g, 13 mmol) in CH_2_Cl_2_ (30 mL), silica gel (200 mesh, 2 g) and PCC (4.2 g, 19.5 mmol) were added. The above reaction mixture was stirred overnight, followed by the addition of ether (50 mL). Then, the solution of crude aldehyde was decanted and purified through flash column chromatography to give 8-oxooctyl isobutyrate **3** (2.6 g, 94%) as a colorless oil. ^1^H-NMR (500 MHz, CDCl_3_) δ 9.76 (t, *J* = 1.8 Hz, 1H), 4.05 (t, *J* = 6.7 Hz, 2H), 2.56–2.50 (m, 1H), 2.42 (td, *J* = 1.7, 7.3 Hz, 2H), 1.64–1.61 (m, 4H), 1.34 (s, 6H), 1.15 (d, *J* = 7.0 Hz, 6H); ^13^C-NMR (125 MHz, CDCl_3_) δ 202.69, 177.22, 64.22, 43.83, 34.02, 29.0, 28.95, 28.55, 25.69, 21.94, 18.99.

### 3.10. (R)-10,14-Dimethylpentadec-8-en-1-yl Isobutyrate (***13***)

Under argon, NaHMDS (1.6 mL, 2.0 M in THF, 3.2 mmol) was added dropwise to a solution of compound **12** (0.8 g, 2.5 mmol) in anhydrous THF (25 mL) at –78 °C. After half an hour, 8-oxooctyl isobutyrate **3** (0.7 g, 3.3 mmol) in THF (10 mL) was added. The reaction mixture was stirred under the same temperature for 3 h, followed by stirring for another 12 h at –50 °C. After the reaction was quenched with saturated aqueous NH_4_Cl at –50 °C, it was extracted with ethyl acetate, washed with brine, dried over anhydrous Na_2_SO_4_, concentrated, and purified by flash chromatography on silica gel (hexanes: ethyl acetate = 30:1) to give compound **13** as a pale-yellow oil (0.6 g, 79%). [α]_D_^25^ + 3.06 (*c* 2.5, CHCl_3_). ^1^H-NMR (500 MHz, CDCl_3_) δ 7.33–7.25 (m, 5H), 5.36–5.08 (m, 2H), 4.49–4.44 (m, 2H), 4.06–4.03 (m, 2H), 3.49–3.39 (m, 2H), 2.65–2.5 (m, 2H), 2.07–1.93 (m, 2H), 1.7–1.43 (m, 4H), 1.3 (s, 8H), 1.16 (d, *J* = 7.0 Hz, 6H), 0.98–0.95 (m, 3H); ^13^C-NMR (125 MHz, CDCl_3_) δ 177.25, 138.76, 138.73, 135.53, 129.05, 128.34, 127.66, 127.61, 127.47, 72.99, 68.82, 64.4, 37.28, 34.08, 29.85, 29.25, 29.19, 28.68, 28.61, 27.43, 25.93, 25.9, 21.51, 19.05.

### 3.11. (S)-10,14-Dimethylpentadecyl Isobutyrate (***1***)

To a solution of compound **13** (0.3 g, 9.3 mmol) in ethanol (15 mL), Pt/C (10%, 6 mg) was added and the atmosphere was exchanged with H_2_. The reaction mixture was then stirred for 24 h and monitored by TLC. After completion, the catalyst was filtered through a Celite pad, and the filtrate was evaporated. The residue was purified by flash chromatography on silica gel (PE/EA = 30:1) to give compound **1** (0.28 g, 93%) as a colorless oil: [α]_D_^25^ − 0.28 (*c* 2.4, CHCl_3_). ^1^H-NMR (500 MHz, CDCl_3_) δ 4.05 (t, *J* = 6.8 Hz, 2H), 2.56–2.5 (m, 1H), 1.65–1.59 (m, 2H), 1.55–1.5 (m, 1H), 1.36–1.19 (m, 17H), 1.16 (d, *J* = 7.1 Hz, 6H), 1.15–1.04 (m, 4H), 0.85 (dd, *J* = 6.7, 13.2 Hz, 9H); ^13^C-NMR (500 MHz, CDCl_3_) δ 177.26, 64.42, 39.4, 37.34, 37.12, 34.08, 32.79, 30.0, 29.62, 29.55, 29.27, 28.68, 28.00, 27.09, 25.93, 24.82, 22.73, 22.64, 19.73, 19.03. HRMS (ESI) *m*/*z* calcd. for C_21_H_43_O_2_^+^ (M + H)^+^: 327.32576, found 327.32599.

## 4. Conclusions

Based on a chiral pool strategy, an efficient synthesis of (*S*)-10,14-dimethylpentadecyl isobutyrate, the sex pheromone of the tea tussock moth, was achieved. The longest linear synthetic step was 10 steps, and the overall yield was 33%. The key step was accomplished by a double Julia coupling. This synthesis will be helpful in research involving the differences between the biological effects of *R*-**1** and *S*-**1**. The scale-up and evaluation of the biological activities of the sex pheromone is currently under investigation.

## Supplementary Materials

Supplementary Materials are available online.
