# Supplementary figures and images for: Synthesis of the Sex Pheromone of the Tea Tussock Moth Based on a Resource Chemistry Strategy
Source: Molecules. 2018 Jun 4;23(6):1347. doi: 10.3390/molecules23061347 (PMC6100017; doi:10.3390/molecules23061347)

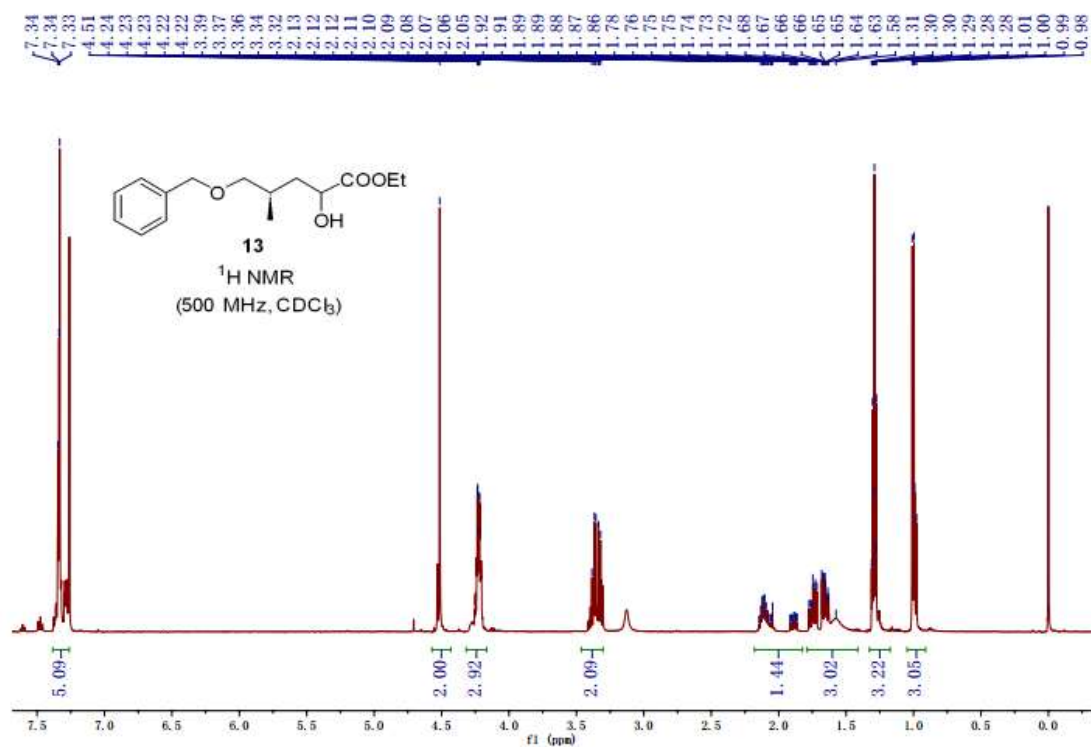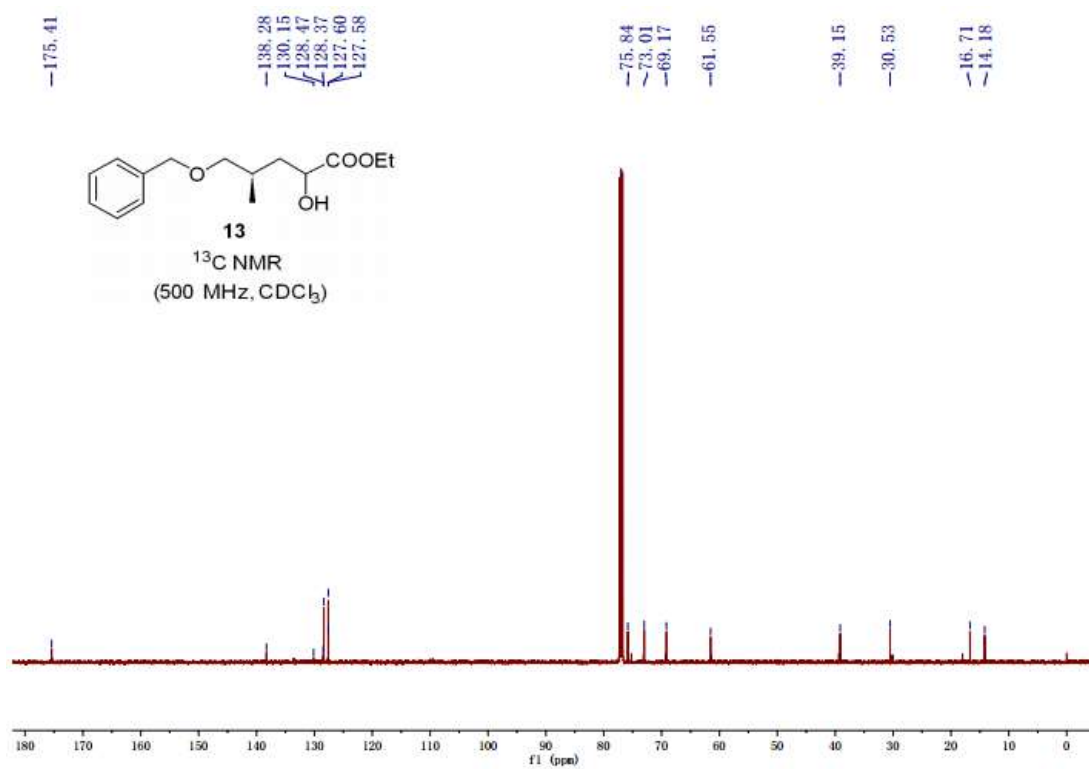

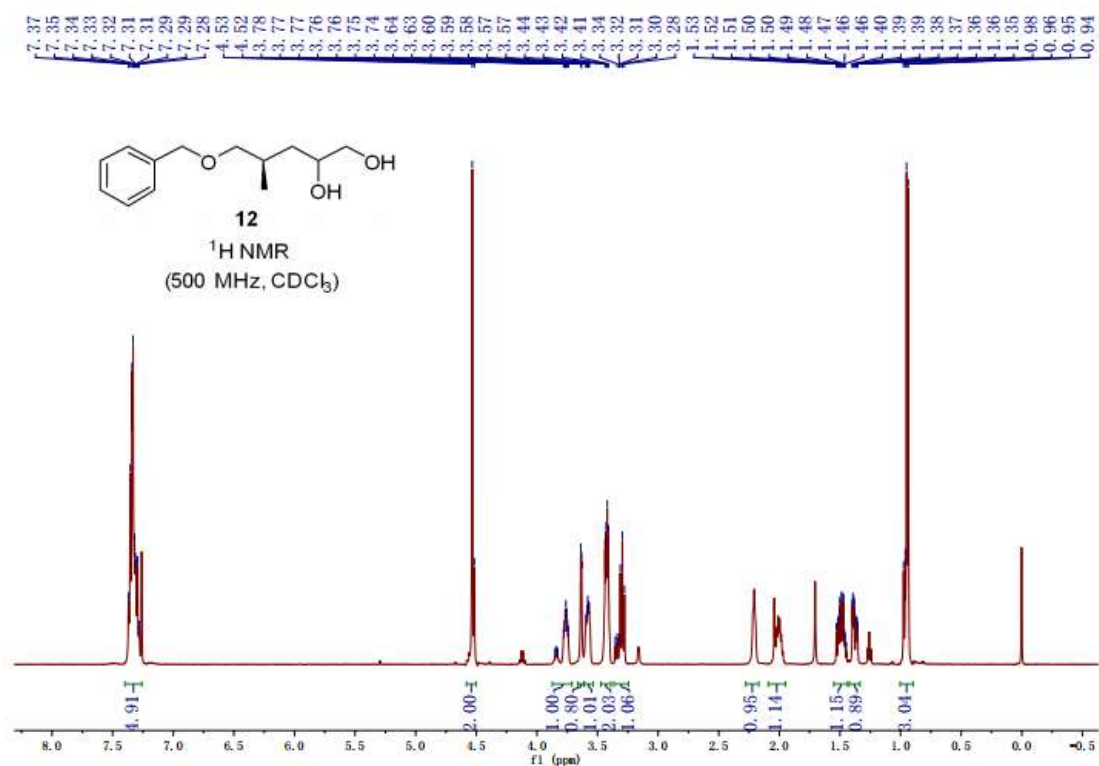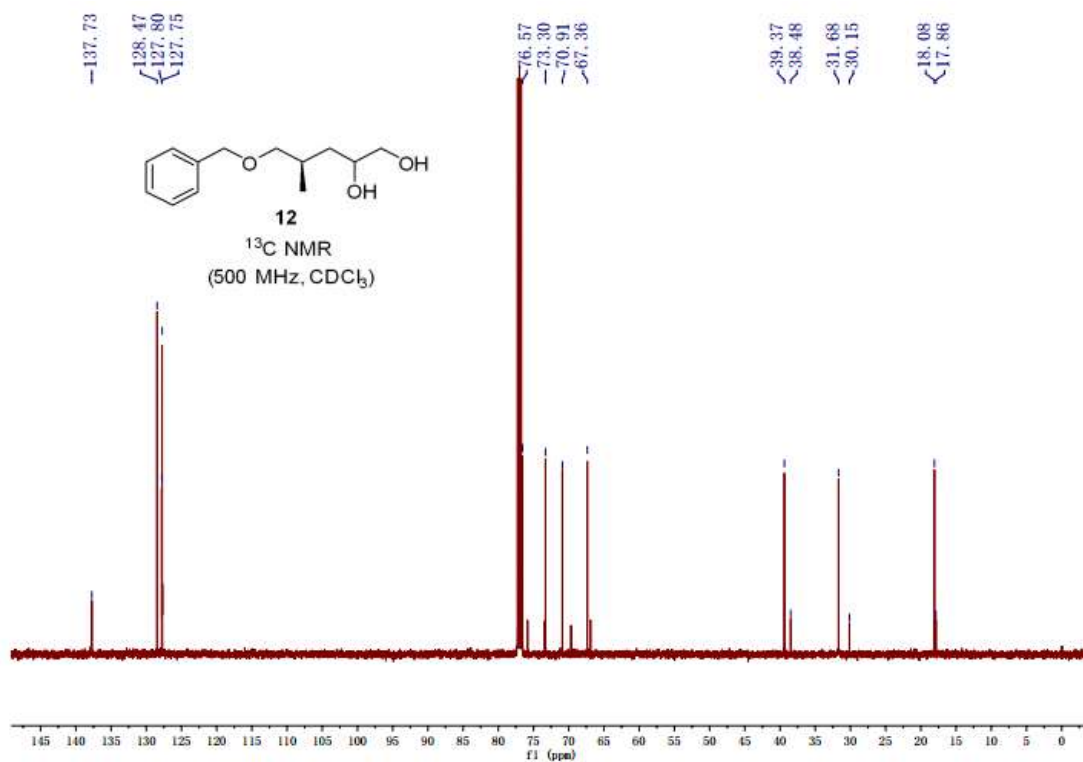

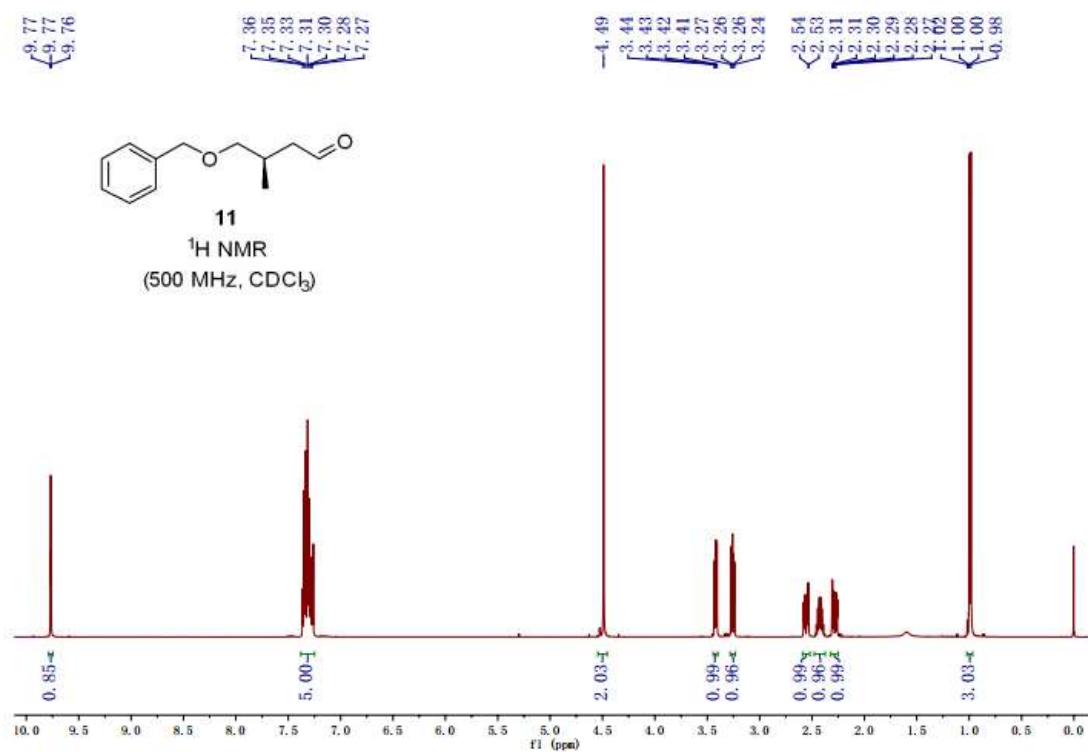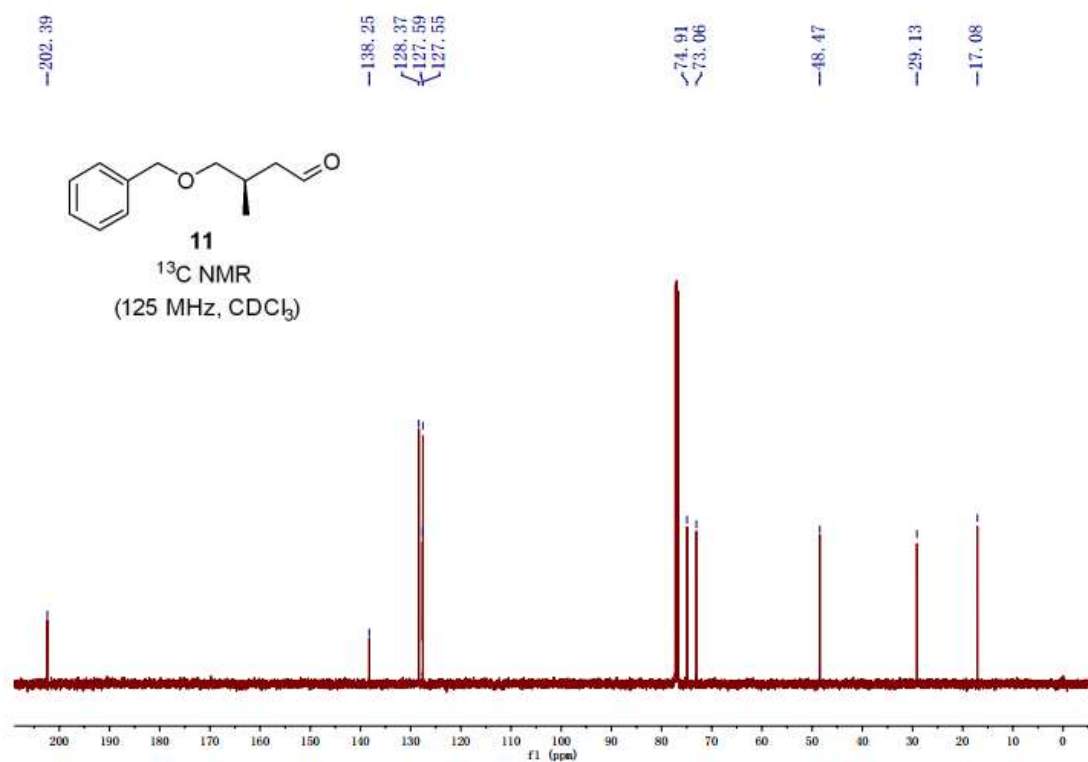

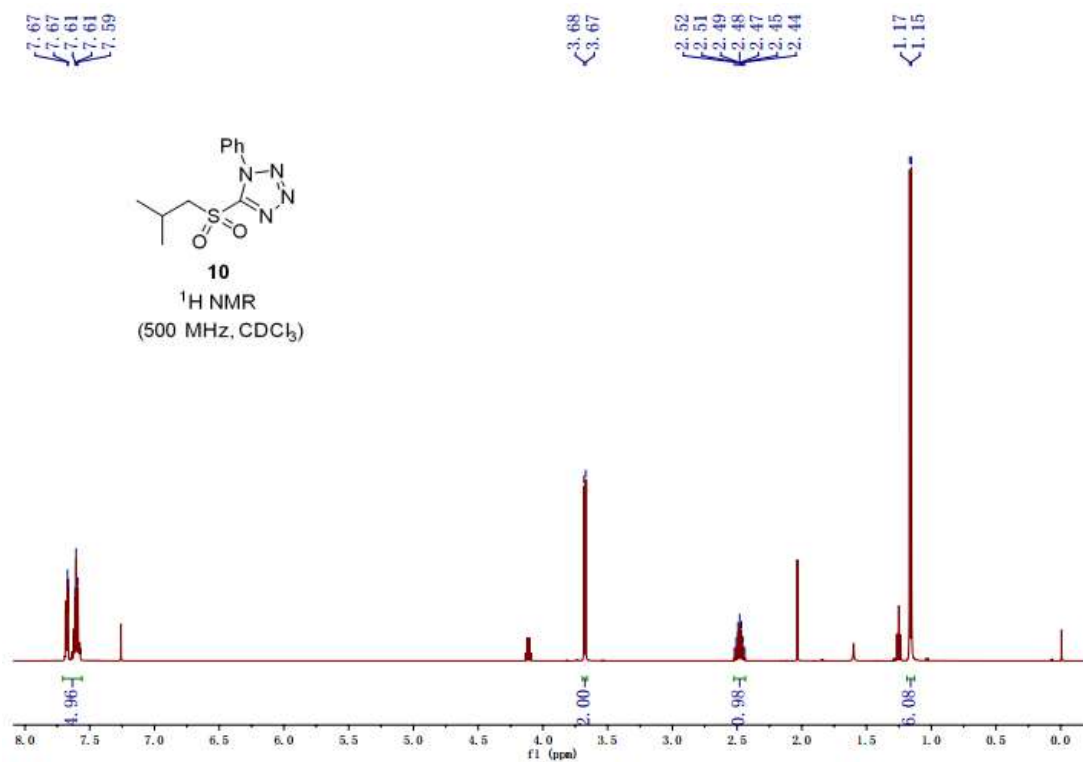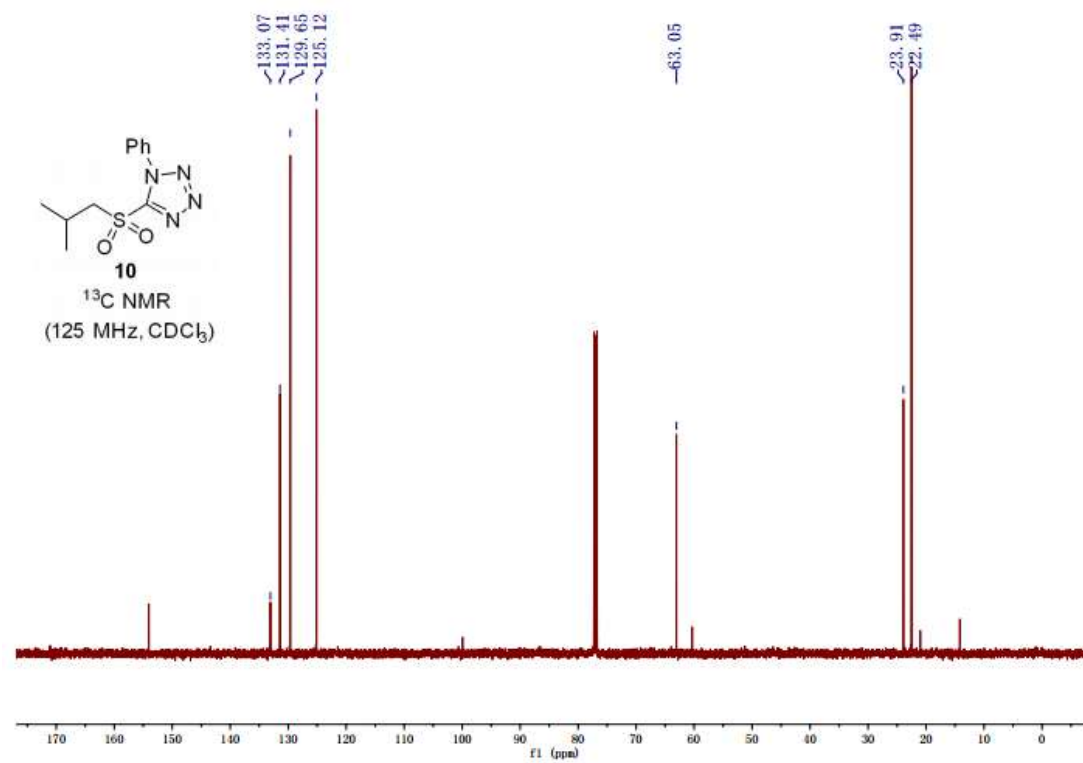

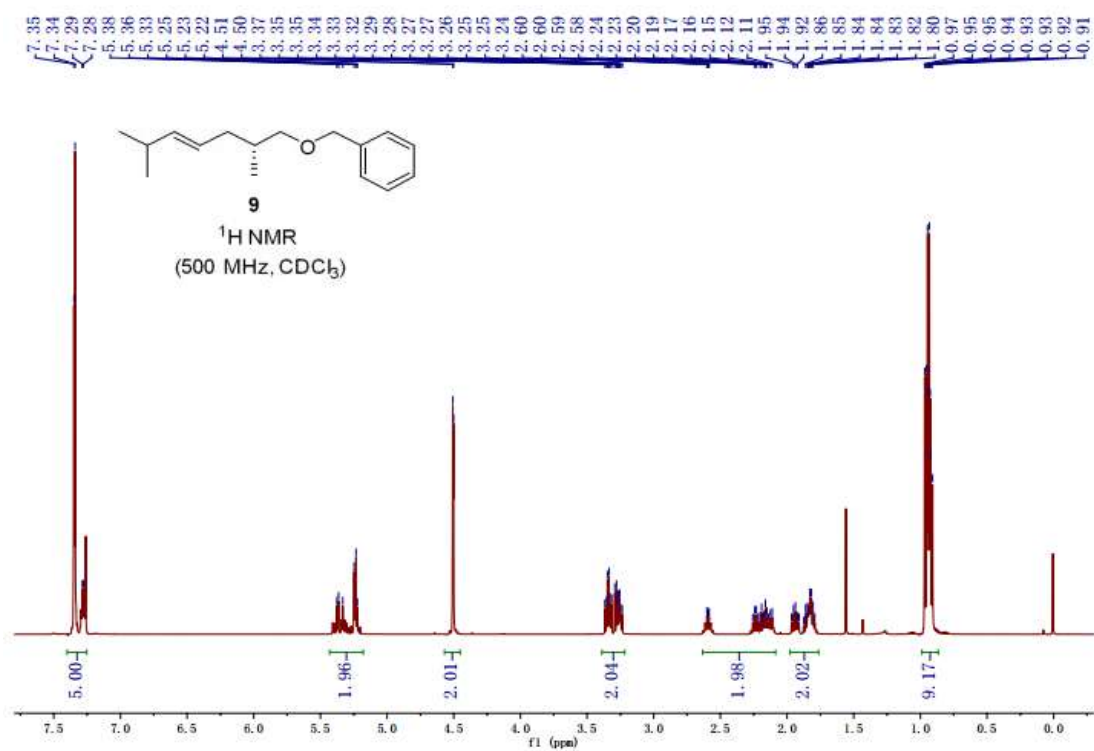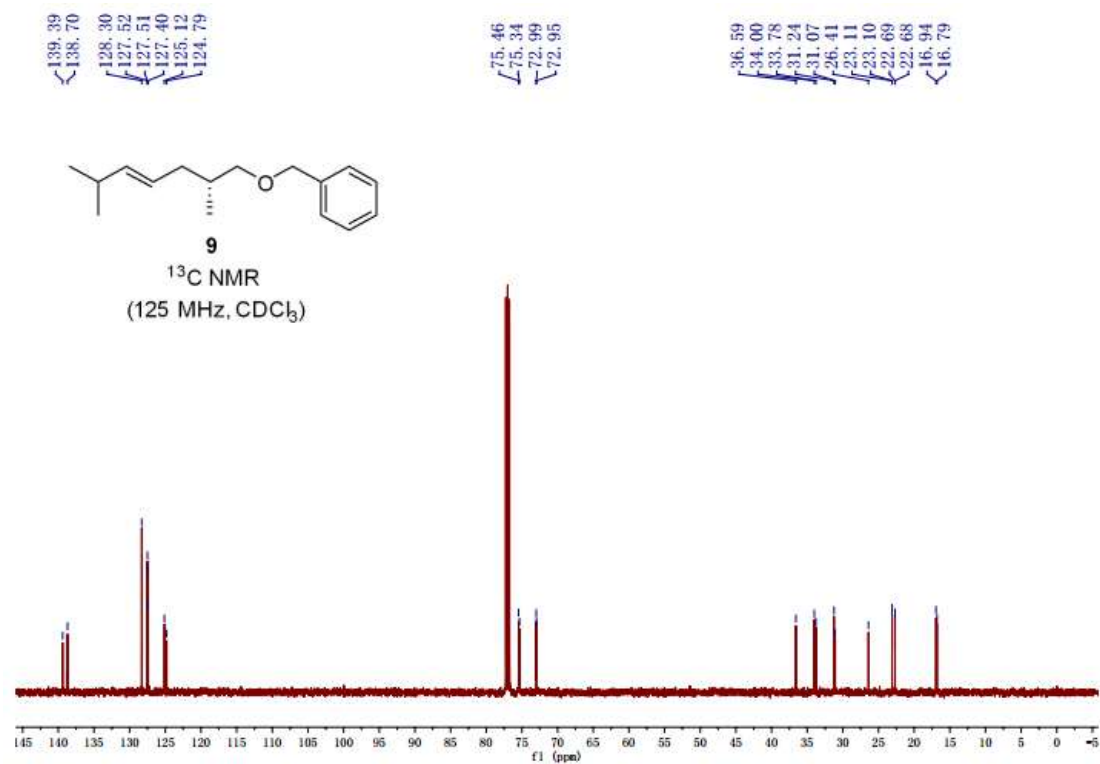

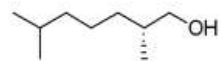

8

$^1\text{H}$  NMR  
(500 MHz,  $\text{CDCl}_3$ )

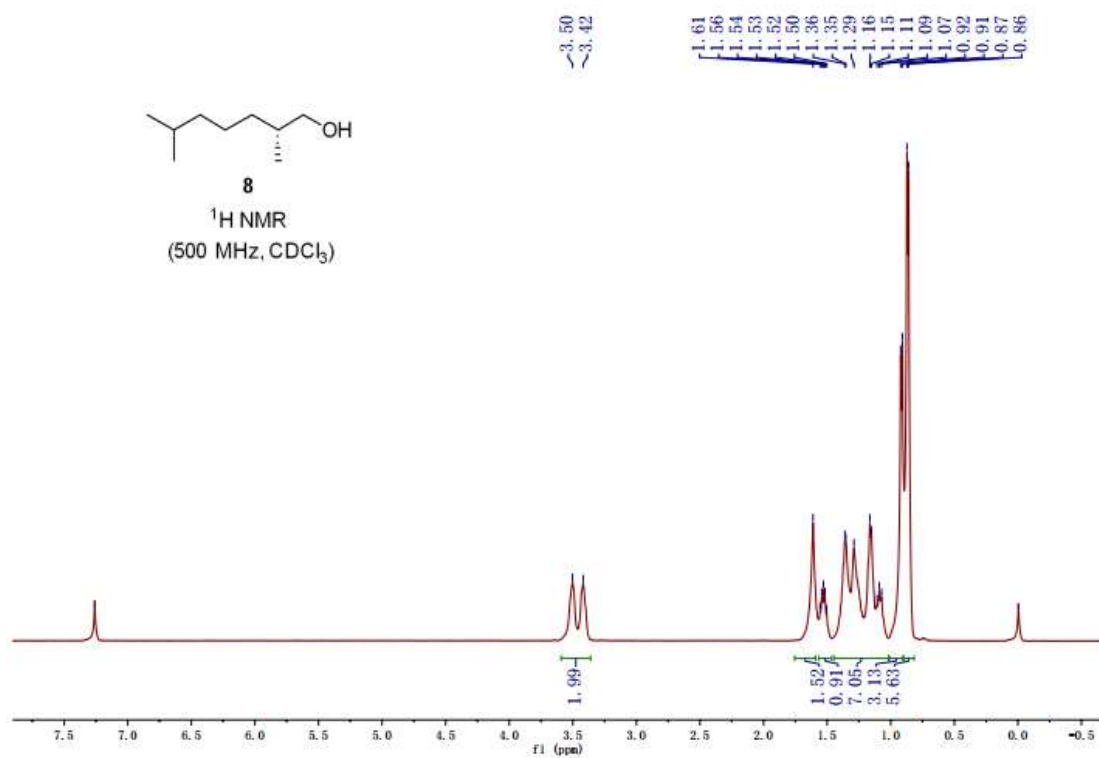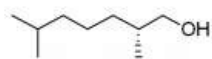

8

$^{13}\text{C}$  NMR  
(125 MHz,  $\text{CDCl}_3$ )

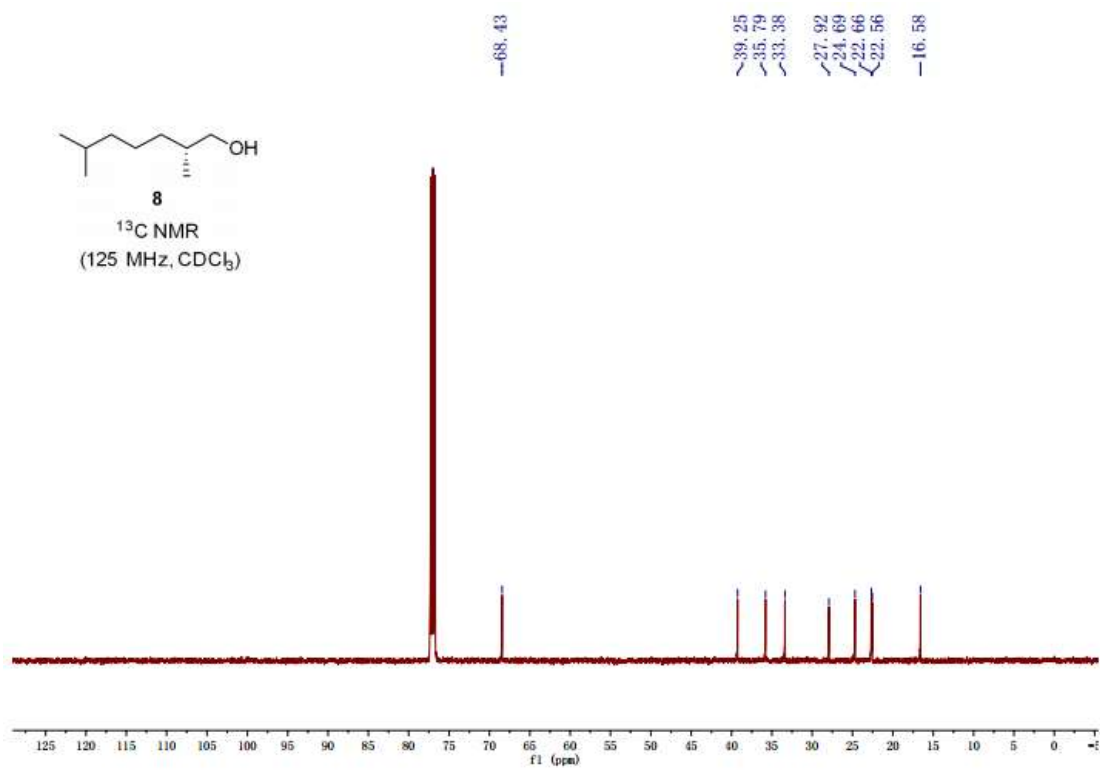

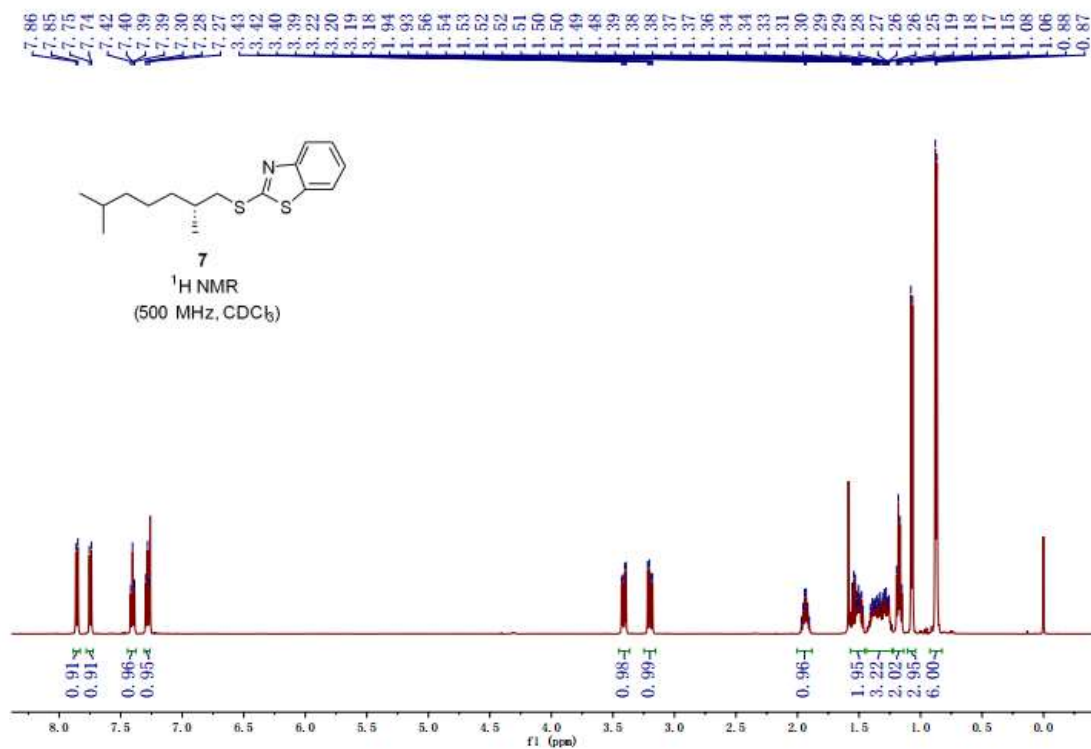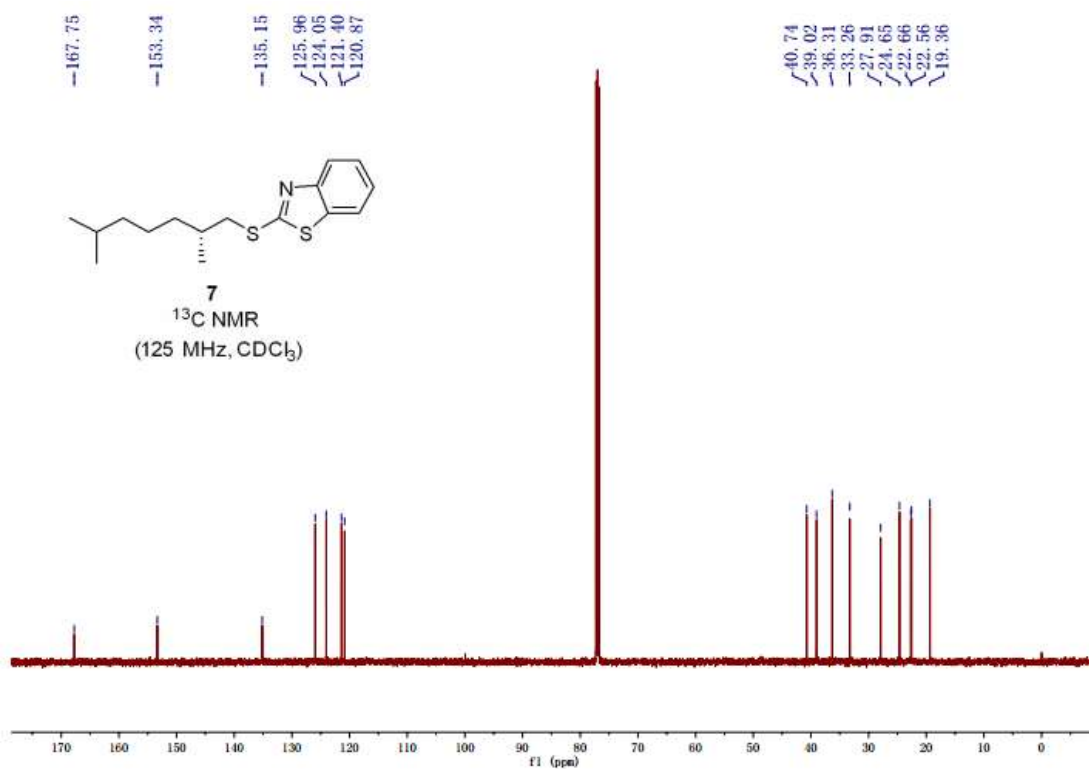

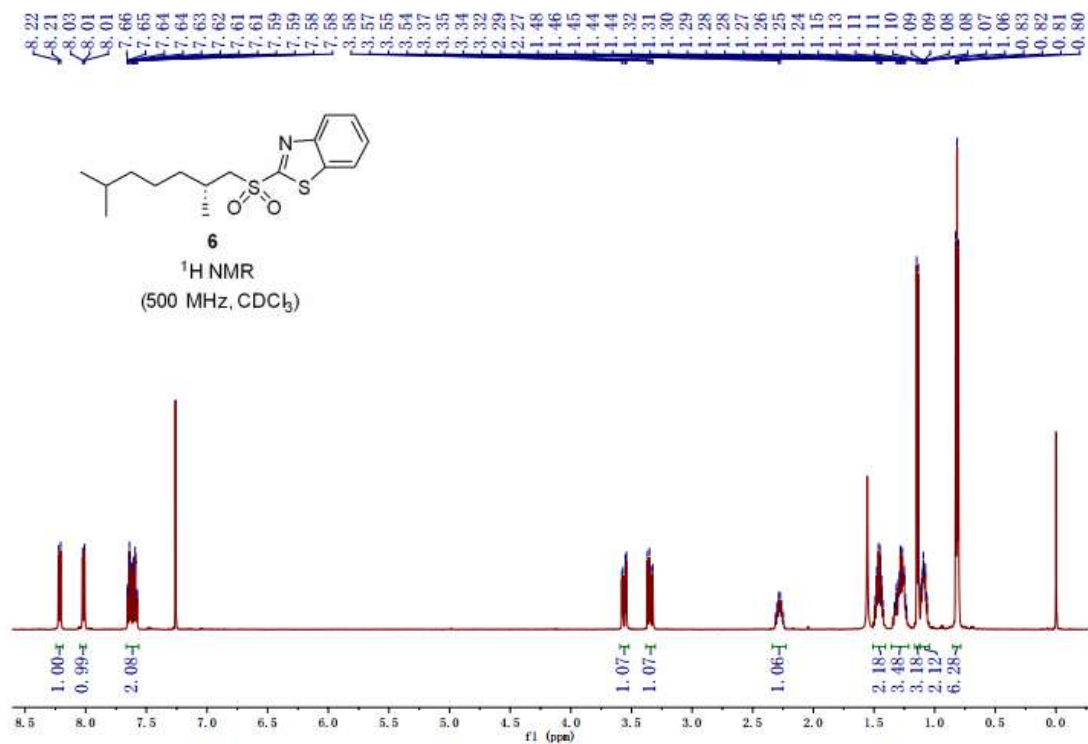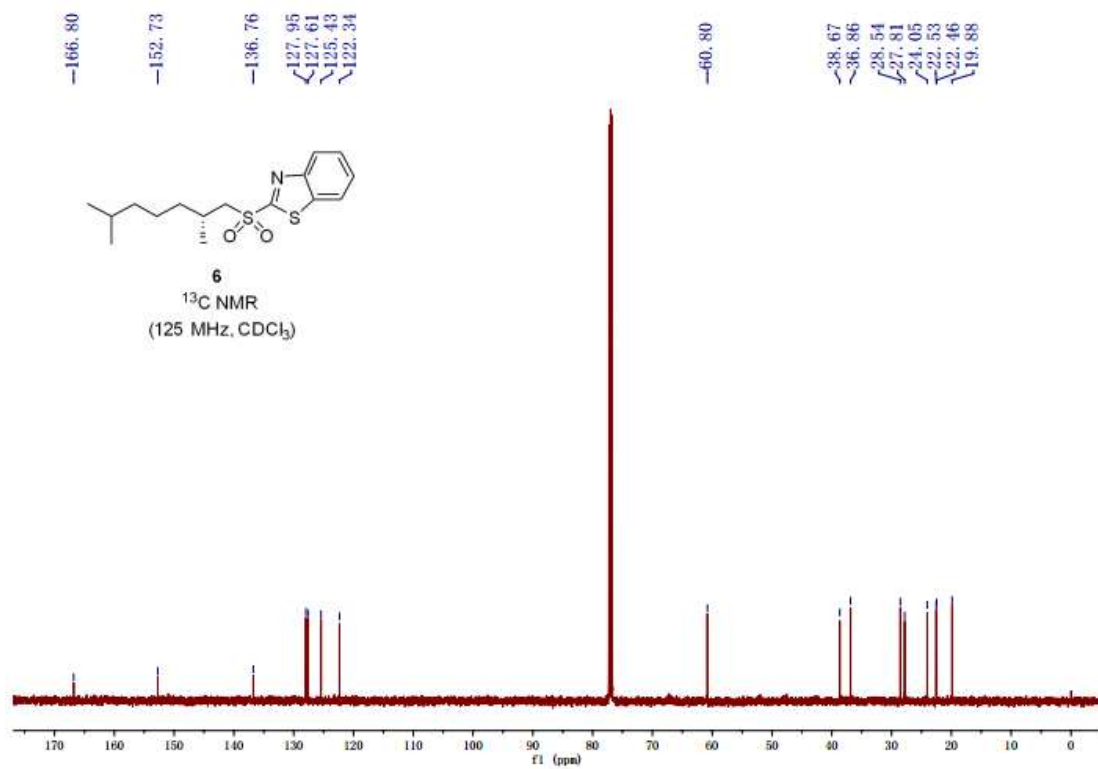

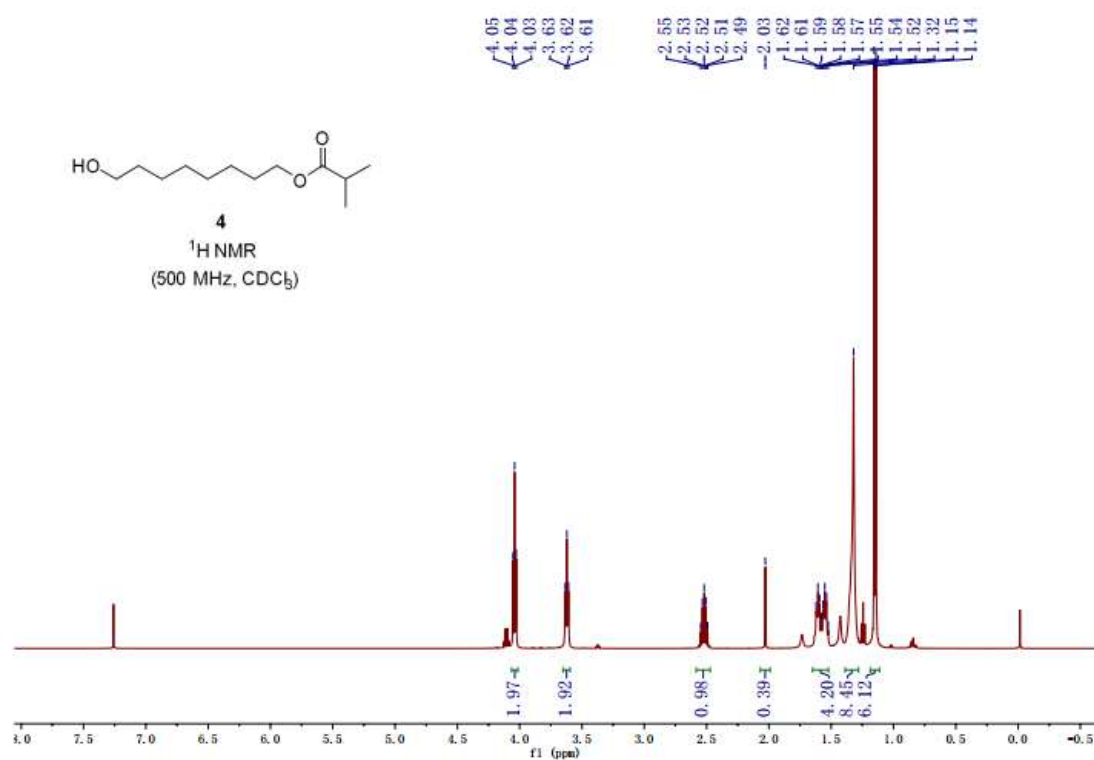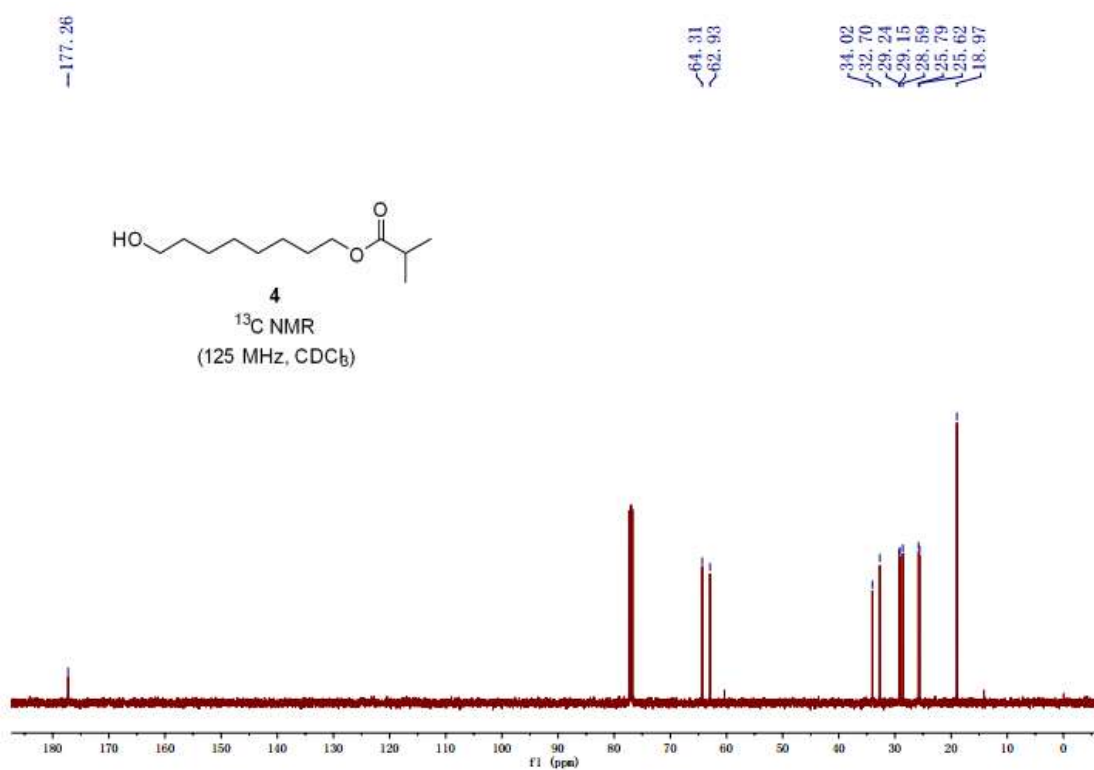

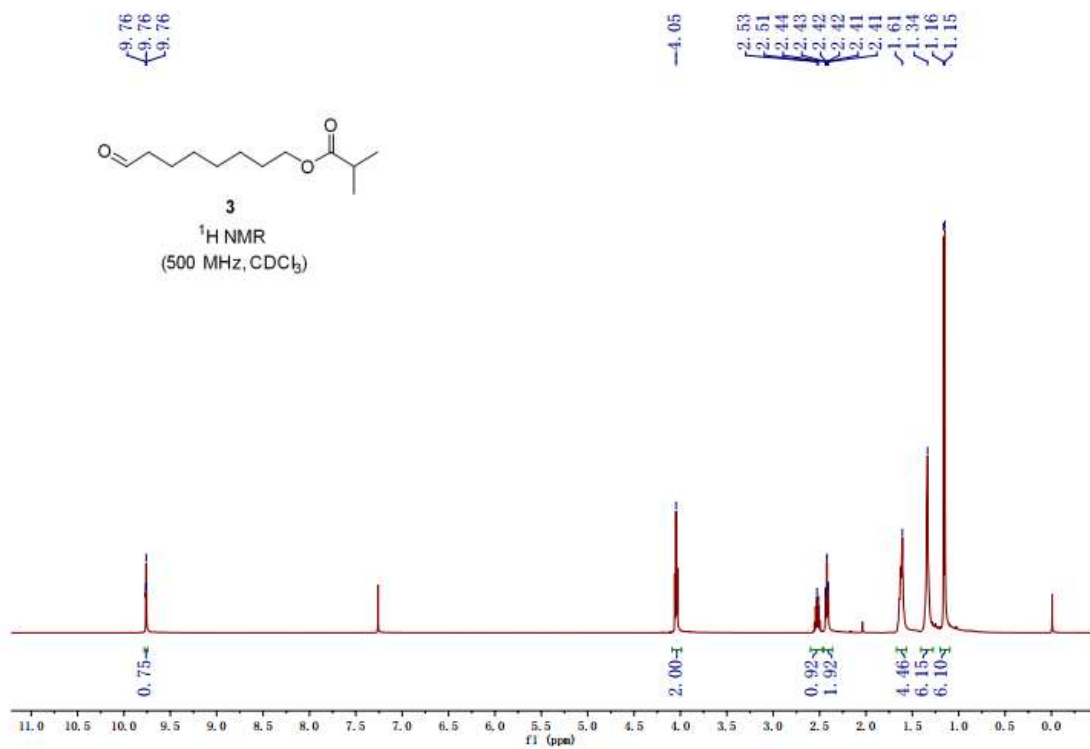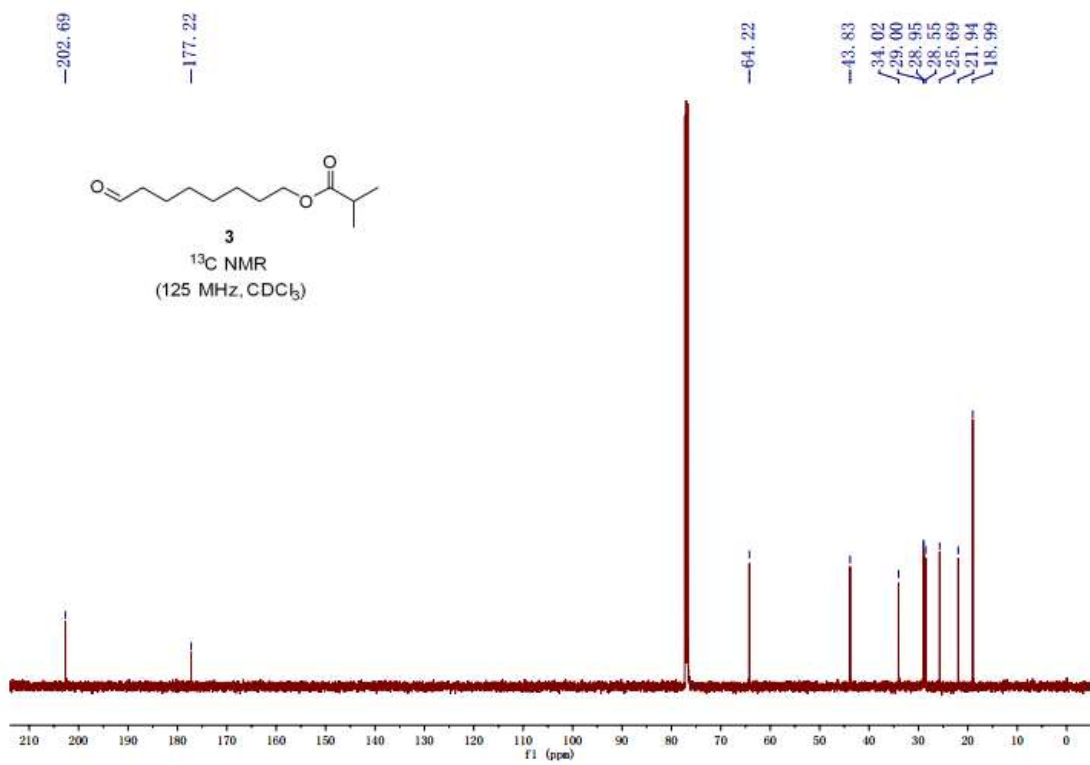

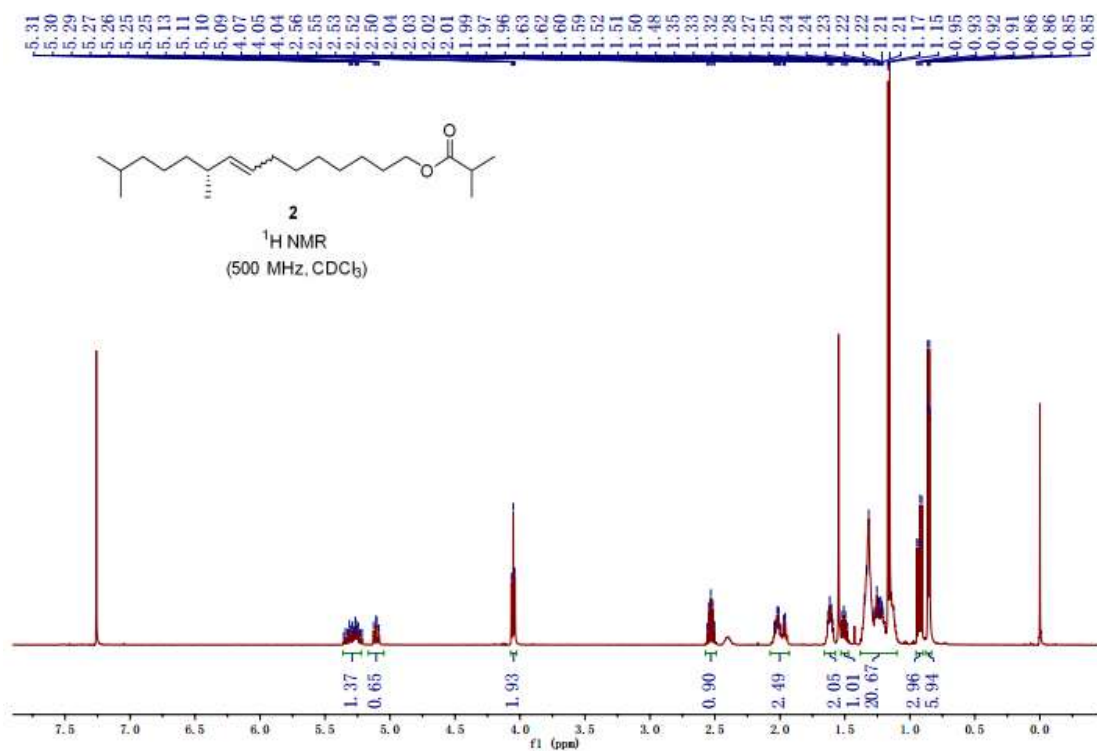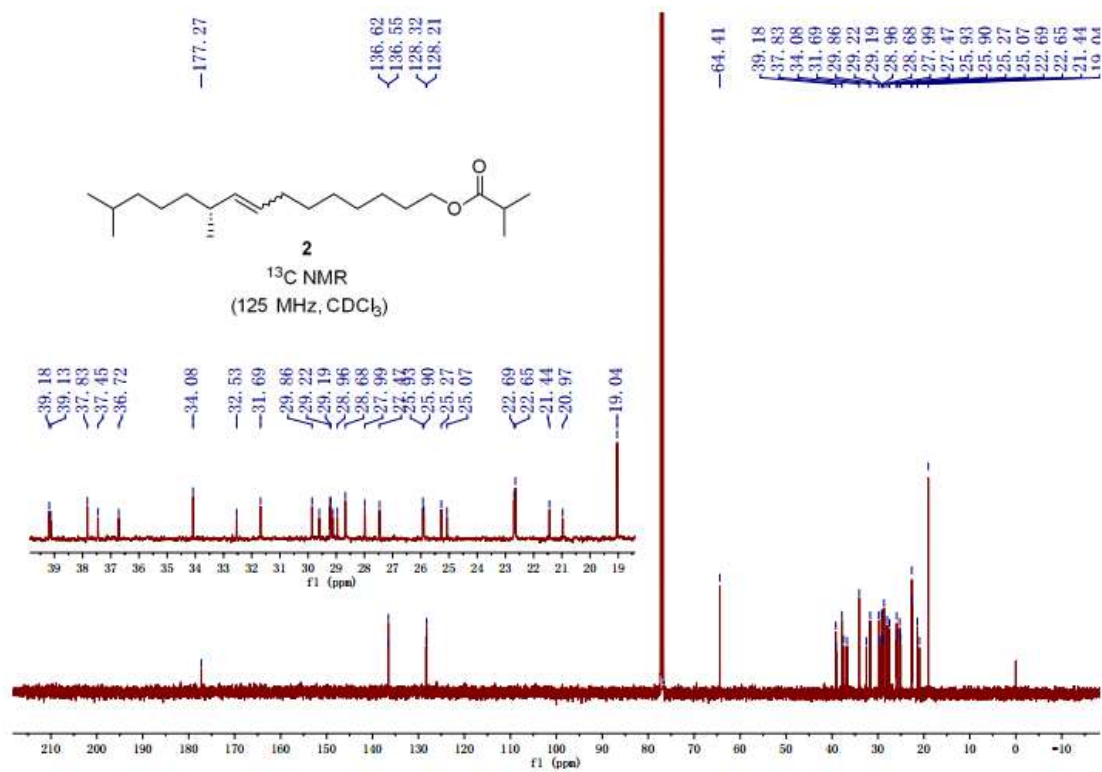

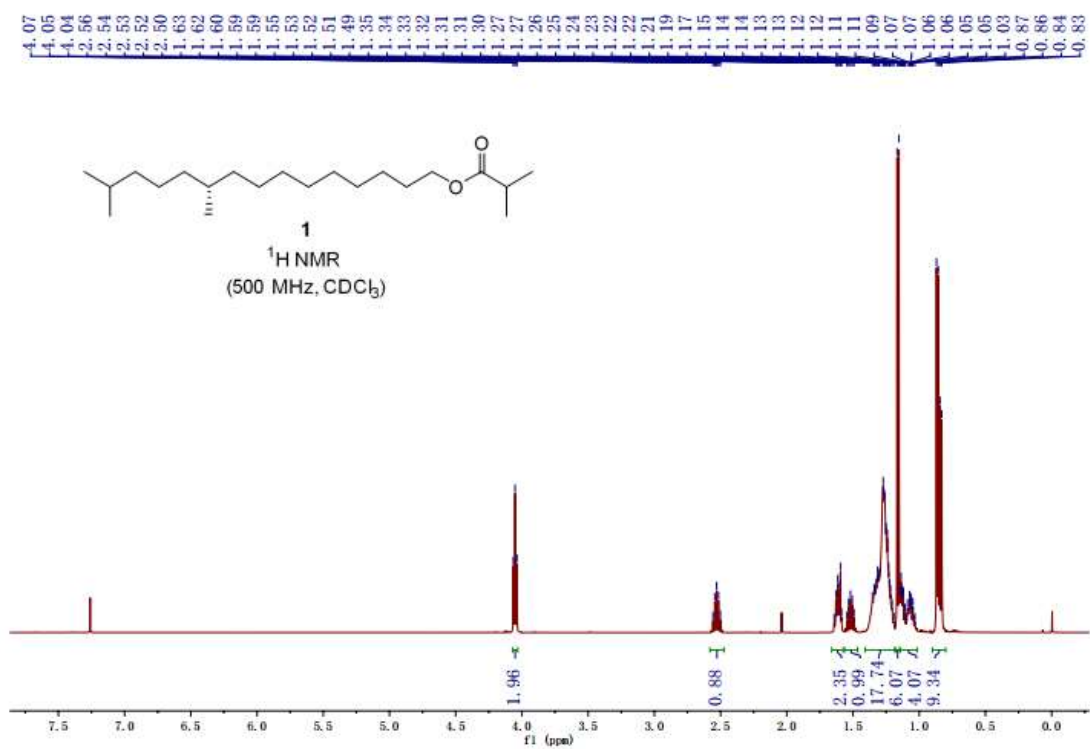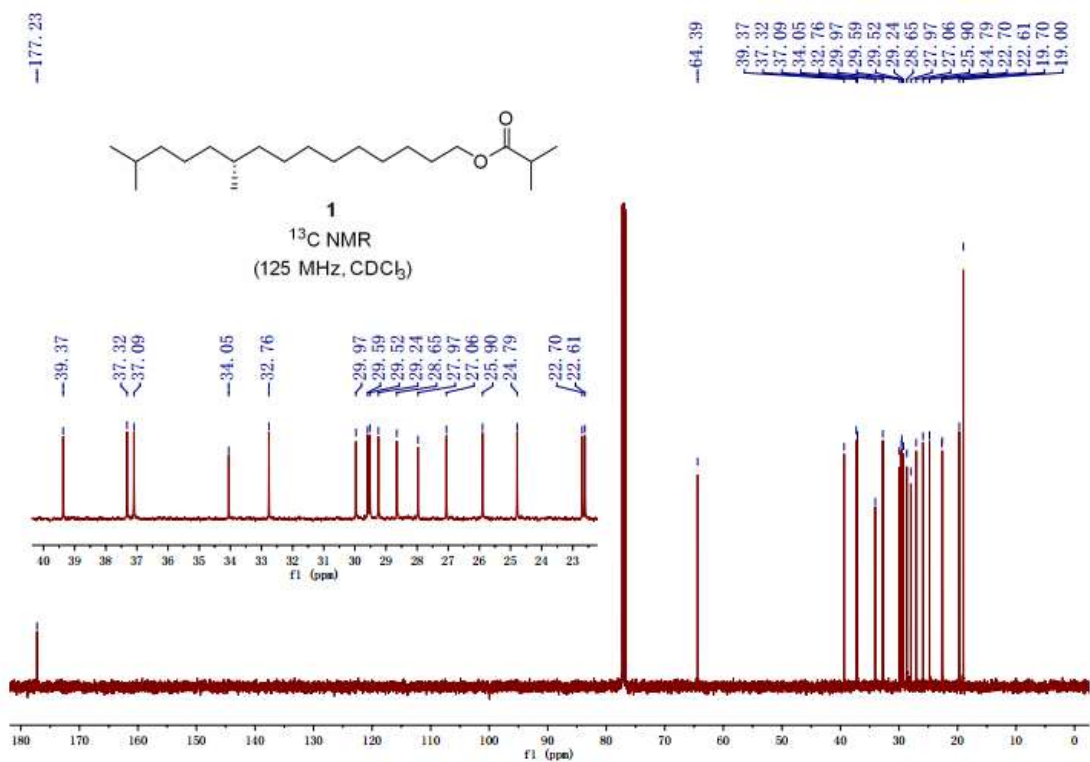

Supplement: Supplementary file 1 [file molecules-23-01347-s001.pdf]
